# Flaxseed oil ameliorates alcoholic liver disease via anti-inflammation and modulating gut microbiota in mice

**DOI:** 10.1186/s12944-017-0431-8

**Published:** 2017-02-22

**Authors:** Xiaoxia Zhang, Hao Wang, Peipei Yin, Hang Fan, Liwei Sun, Yujun Liu

**Affiliations:** 1 College of Biological Sciences and Biotechnology, Beijing Forestry University, Qinghua Donglu No35, Haidian District, Beijing, 100083 China; 2 Ningxia Medical University, Yinchuan, 750004 Ningxia China

**Keywords:** Flaxseed oil, ALD, Anti-inflammation, Gut microbiota

## Abstract

**Background:**

Alcoholic liver disease (ALD) represents a chronic wide-spectrum of liver injury caused by consistently excessive alcohol intake. Few satisfactory advances have been made in management of ALD. Thus, novel and more practical treatment options are urgently needed. Flaxseed oil (FO) is rich in α-linolenic acid (ALA), a plant-derived n-3 polyunsaturated fatty acids (PUFAs). However, the impact of dietary FO on chronic alcohol consumption remains unknown.

**Methods:**

In this study, we assessed possible effects of dietary FO on attenuation of ALD and associated mechanisms in mice. Firstly, mice were randomly allocated into four groups: pair-fed (PF) with corn oil (CO) group (PF/CO); alcohol-fed (AF) with CO group (AF/CO); PF with FO group (PF/FO); AF with FO group (AF/FO). Each group was fed modified Lieber-DeCarli liquid diets containing isocaloric maltose dextrin a control or alcohol with corn oil and flaxseed oil, respectively. After 6 weeks feeding, mice were euthanized and associated indications were investigated.

**Results:**

Body weight (BW) was significantly elevated in AF/FO group compared with AF/CO group. Dietary FO reduced the abnormal elevated aspartate aminotransferase (AST) and alanine aminotransferase (ALT) levels in chronic ethanol consumption. Amelioration of these parameters as well as liver injury via HE staining in dietary FO supplementation in ALD demonstrated that dietary FO can effectively benefit for the protection against ALD. To further understand the underlying mechanisms, we investigated the inflammatory cytokine levels and gut microbiota. A series of inflammatory cytokines, including TNF-α, IL-1β, IL-6 and IL-10, were determined. As a result, TNF-α, IL-1β and IL-6 were decreased in AF/FO group compared with control group; IL-10 showed no significant alteration between AF/CO and AF/FO groups (*p* > 0.05). Sequencing and analysis of gut microbiota gene indicated that a reduction of *Porphyromonadaceae* and *Parasutterella*, as well as an increase in *Firmicutes* and *Parabacteroides*, were seen in AF group compared with PF control. Furthermore, dietary FO in ethanol consumption group induced a significant reduction in *Proteobacteria* and *Porphyromonadaceae* compared with AF/CO group.

**Conclusion:**

Dietary FO ameliorates alcoholic liver disease via anti-inflammation and modulating gut microbiota, thus can potentially serve as an inexpensive interventions for the prevention and treatment of ALD.

**Electronic supplementary material:**

The online version of this article (doi:10.1186/s12944-017-0431-8) contains supplementary material, which is available to authorized users.

## Background

Alcoholic liver disease (ALD) represents a chronic wide-spectrum of liver injury caused by consistently excessive alcohol intake, ranking major causes of morbidity and mortality worldwide among people who abuse alcohol [1]. ALD includes a histological spectrum of liver injure ranging from simple steatosis to hepatitis characterized by inflammation, with potential progression to fibrosis and cirrhosis. Hepatitis, with an occurrence of approximately 10 to 35% in chronic drinkers and responsible for more than 1/3 significant morbidity and mortality, has been thought to play a crucial role in reversible pathological process of ALD [2–4]. Up to now, few satisfactory advances have been made in management of ALD, except abstinence from alcohol [4, 5]. Thus, novel and more practical treatment options are urgently needed.

Gut microbiota play a crucial role in progression and pathogenesis of ALD. Accumulating evidence has revealed that gut microbiota is closely associated with liver in ALD as the gut-liver axis [6, 7]. Impairment of gut microbiota homeostasis in ALD induces proliferation of gram negative pathogenic bacteria, which generate lipopolysaccharide (LPS) and translocate to liver tissue as a trigger for hepatitis by binding to TLR-4 (Toll-like receptor-4) on macrophages and neutrophils. Moreover, Campos Canesso et al. showed that the administration of alcohol to germ-free mice is associated to the absence of liver inflammation and injury, indicating that alcohol alone is not sufficient for the development of liver disease, and that the presence of microbiota alterations is also necessary [8]. Thus, modulation of gut microbiota dysbiosis could attenuate hepatic injury in ALD [3, 9].

Flaxseed oil (FO) is rich in plant-derived omega-3 (n-3) polyunsaturated fatty acids (PUFAs), mainly α-linolenic acid (ALA, 18:3 n-3). Clinical studies reported that a low levels of n-3PUFAs in serum and liver tissue is a common characteristic of ALD patients [10, 11]. Dietary FO prevented against acute alcoholic hepatic steatosis via ameliorating lipid homeostasis at adipose tissue-liver axis in mice [11]. However, the impact of dietary FO on inflammation and gut micorbiota in chronic ALD remains unknown.

In the present study, we assessed effects of dietary FO on attenuation of ALD and associated mechanisms in mice. Results of the study may contribute to understanding the role played by FO in ALD and the complexity of the interplay among the diet, gut microbiota, inflammation and ALD.

## Methods

### Animals and diet

Sixty male C57BL/6 J mice (8 weeks old) were obtained from Vital River Laboratory Animal Technology Co. Ltd., Beijing, China. The animals were housed in individual cages in a temperature-controlled (22 ± 1 °C), light-cycled (12-h light/dark cycle) room.

All liquid diets for mice feeding were purchased from TROPHIC Animal Feed High-tech Co., Ltd., Nantong, China.

### Experimental design

After an 1-week period of acclimation to the control liquid diet, maleC57BL/6 J mice (*n* = 60, 8 weeks old) were fed the modified Lieber-DeCarli liquid diets as previously described [11]. Briefly, mice were randomly allocated into four groups (15 animals/group): (a) pair-fed (PF) with corn oil (CO) group (PF/CO), mice were fed modified Lieber-DeCarli CO liquid diets containing isocaloric maltose dextrin as CO control; (b) alcohol-fed (AF) with CO group (AF/CO), mice were fed ethanol-containing modified Lieber-DeCarli CO liquid diets; (c) PF with flaxseed oil (FO) group (PF/FO), mice were fed modified Lieber-DeCarli FO liquid diets containing isocaloric maltose dextrin as FO control; (d) AF with FO group (AF/FO), mice were fed ethanol-containing modified Lieber-DeCarli FO liquid diets. Mice in AF groups were fed the modified Lieber-DeCarli liquid diets containing ethanol with an energy composition of 18% protein, 19% carbohydrate, 35% fat and 28% ethanol, whereas animals in the PF groups were fed the modified Lieber-DeCarli liquid diets, in which, isocaloric maltose dextrin (carbohydrate) replaced ethanol, and 35% of the total calories were provided by either corn oil (rich in n-6 PUFAs) or flaxseed oil (rich in n-3 PUFAs). Components of the liquid diets and the fatty acid composition of dietary fats are shown in Additional file 1 (Table S1) and Additional file 2 (Table S2), respectively. Groups (a) and (c) were the pair-fed controls for groups (b) and (d), respectively. Liquid diets were freshly prepared from powder daily according to the manufacturer’s instruction. Average daily volume of liquid intake per mouse was monitored and calculated in AF groups. Mice in PF groups consume equal amounts of diets. After 6 weeks of feeding, mice were then euthanized and associated indications were investigated. Blood samples were collected in ethylene diamine tetraacetic acid (EDTA)-containing tubes and centrifuged (1200 × g for 15 min) to obtain plasma samples. All plasma samples were stored at −80 °C for further analysis.

### Determination of plasma AST and ALT levels

As biochemical indicators of liver function, plasma aspartate aminotransferase (AST) and alanine aminotransferase (ALT) activities in each group were respectively determined using AU400 automatic biochemical analyzer (Olympus, Japan).

### Determination of plasma endotoxin

Plasma LPS levels in each mouse/group were measured with limulus amebocyte lysate kit (Xiamen Bioendo Technology Co.Ltd, Xiamen, China) according to the manufacturer’s instructions.

### HE staining

After mice sacrifice, liver tissues were immediately fixed with formalin and processed with hematoxylin-eosin (HE) staining to evaluate liver damage including hepatocyte fatty change, inflammatory cells, degeneration and necrosis.

### ELISA assays

Liver tissues (0.5 g) were homogenized in 1.5 ml ice-cold 50 mM Tris buffer (pH7.2, Tris with 1% Triton-X 100 and 0.1% protease inhibitor) and shaken on ice for 90 min. Then the homogenates were centrifuged at 3,000 × g for 15 min. Supernatants were collected for determination of tumor necrosis factor (TNF)-α, IL (interleukin)-1β, IL-6 and IL-10 concentrations. Measurements of each cytokine level in plasma or the supernatants of liver tissues were performed by enzyme linked immunosorbent assay (ELISA) according to the manufacturer’s instructions (e-Bioscience, CA, USA).

### Gut microbiota analysis

The fecal microbial 16S rRNA gene sequencing and analysis were investigated as previously described [12]. After 6 weeks feeding, five mice per group were randomly selected and transferred to fresh sterilized cages. The fresh feces of each mouse was respectively collected, immediately frozen in liquid nitrogen, and then stored at −80 °C until DNA extraction.

Microbial DNA was extracted from 200 mg feces samples as previously described [13]. Briefly, this sample (200 mg) was resuspended in 4 ml of 4 M guanidine thiocyanate–0.1 M Tris (pH7.5) and 600 μl of 10% N-lauroyl sarcosine. The feces was ground with a mortar on ice, 250 μg of the ground material was transferred to a 2-ml screw-cap polypropylene microcentrifuge tube, and the remaining material was frozen. After addition of 500 μl of 5% N-lauroyl sarcosine 0.1 M phosphate buffer (pH8.0), the 2 ml tube was incubated at 70 °C for 1 h. One volume (750 μl) of 0.1 mm diameter silica beads (Sigma) previously sterilized by autoclaving was added, and the tube was shakenat maximum speed for 10 min in a Vibro shaker (Retsch). Polyvinylpolypyrrolidone (15 mg) was added to the tube, which was vortexed and centrifuged for 3 min at 12,000 × g. After recovery of the supernatant, the pellet was washed with 500 μl of TENP (50 mM Tris [pH8], 20 mM EDTA [pH8], 100 mM NaCl, 1% polyvinylpolypyrrolidone) and centrifuged for 3 min at 12,000 × g, and the new supernatant was added to the first supernatant. The washing step was repeated three times. Pooled supernatants (about 2 ml) were briefly centrifuged to remove particles and then split into two 2 ml tubes. Nucleic acids were precipitated by the addition of 1 volume of isopropanol for 10 min at room temperature and centrifuged for 15 min at 20,000 × g. Pellets were resuspended and pooled in 450 μl of 100 mM phosphate buffer (pH8) and 50 μl of 5 M potassium acetate. The tube was placed on ice for 90 min and centrifuged at 16,000× g for 30 min. The supernatant was transferred to a new tube containing 20 μl of RNase (1 mg/ml) and incubated at 37 °C for 30 min. Nucleic acids were precipitated by addition of 50 μl of 3 M sodium acetate and 1 ml of absolute ethanol. The tube was incubated for 10 min at room temperature, and nucleic acids were recovered by centrifugation at 20,000 × g for 15 min. The DNA pellet was finally washed with 70% ethanol, dried, and resuspended in 400 μl TE buffer. DNA concentration and purity were analyzed by Nanodrop (Thermo). Size distribution (predominantly around 20 kb) were estimated by electrophoresis (Additional file 3: Figure S1). Extracted DNA was stored at −20 °C until use.

Sequences involving V3 and V4 16S rDNA hypervariable regions were amplified by TranStart FastPfu DNA Polymerase (TransGen Biotech, China) using the following primers (5’ to 3’): 341 F-CCTACGGGNGGCWGCAG, 805R-GACTACHVGGGTATCTAATCC. PCR products were analyzed and separated by electrophoresis on 2% agarose gel (containing SYB green), then purified with Qiagen Gel Extraction Kit (Qiagen, Germany). Sequencing libraries were generated using TruSeq DNA PCR manufacturer’s instructions and index codes were added. The library was sequenced and analyzed using an Illumina HisSeq2500 platform by Shanghai Tai Chang gene technology co., LTD., China

### Statistical analysis

All data were analyzed using Prism 5.0 (GraphPad Software Inc., CA, USA). Results were represented as mean ± SEM. Two-way analysis of variance (ANOVA) followed by the Turkey multiple-comparison test was used to determine statistical difference between experimental groups. Results were considered significant at *P* < 0.05.

## Results

### Routine parameters of mice in diverse dietary groups

There was no significant difference in initial body weight (BW) among four groups. However, after 6 weeks feeding, the final BW in AF/CO group was significantly decreased, compared with that in paired PF/CO group (*P* < 0.01) or AF/FO group (*P* < 0.01). The final BW in AF/FO showed no change compared with PF/FO. These results demonstrated that flaxseed oil maintained the BW during chronic ethanol feeding. Liver weight in AF group (AF/CO group and AF/FO group) was significantly elevated comparing to that in PF group (PF/CO group and PF/FO group) (Table 1). Similarly, the ratio of liver-to-body weight in alcohol exposure group regardless of dietary fat was significantly increased compared with that in no ethanol pair-fed group. In addition, the plasma AST and ALT levels in AF/CO group were significantly elevated by 2.5-fold (185.9 ± 13.3 vs. 74.8 ± 8.6) and 2-fold (104.8 ± 11.4 vs. 52.6 ± 5.9) compared with that in pair-fed PO/CO group, respectively. However, these AST and ALT elevations in AF/CO group were effectively suppressed by dietary FO administration in AF/FO group (185.9 ± 13.3 vs. 109.7 ± 7.2, 104.8 ± 11.4 vs. 75.2 ± 6.1) (Table 1).

**Table 1:** Routine parameters of mice in diverse dietary groups in ALD

Measurements	PF/CO	AF/CO	PF/FO	AF/FO	Two-way ANOVA		
					Ethanol	Oil	Interaction
Body weight, g	26.15 ± 0.27	23.99 ± 0.29	26.34 ± 0.33	26.57 ± 0.28	<0.0001	0.0019	0.0002
Liver weight, g	0.89 ± 0.03	1.25 ± 0.04	1.00 ± 0.02	1.44 ± 0.04	<0.0001	<0.0001	0.2722
LW/BW, %	3.40 ± 0.11	5.21 ± 0.14	3.80 ± 0.06	5.42 ± 0.14	<0.0001	<0.0001	0.0027
AST, U/L	74.8 ± 8.6	185.9 ± 13.3	68.4 ± 6.7	109.7 ± 7.2	<0.0001	<0.0001	<0.0001
ALT, U/L	52.6 ± 5.9	104.8 ± 11.4	47.6 ± 8.2	75.2 ± 6.1	<0.0001	<0.0001	<0.0001

### Dietary FO attenuated hepatic histopathological injury and reduced plasma LPS levels

**Fig. 1:**
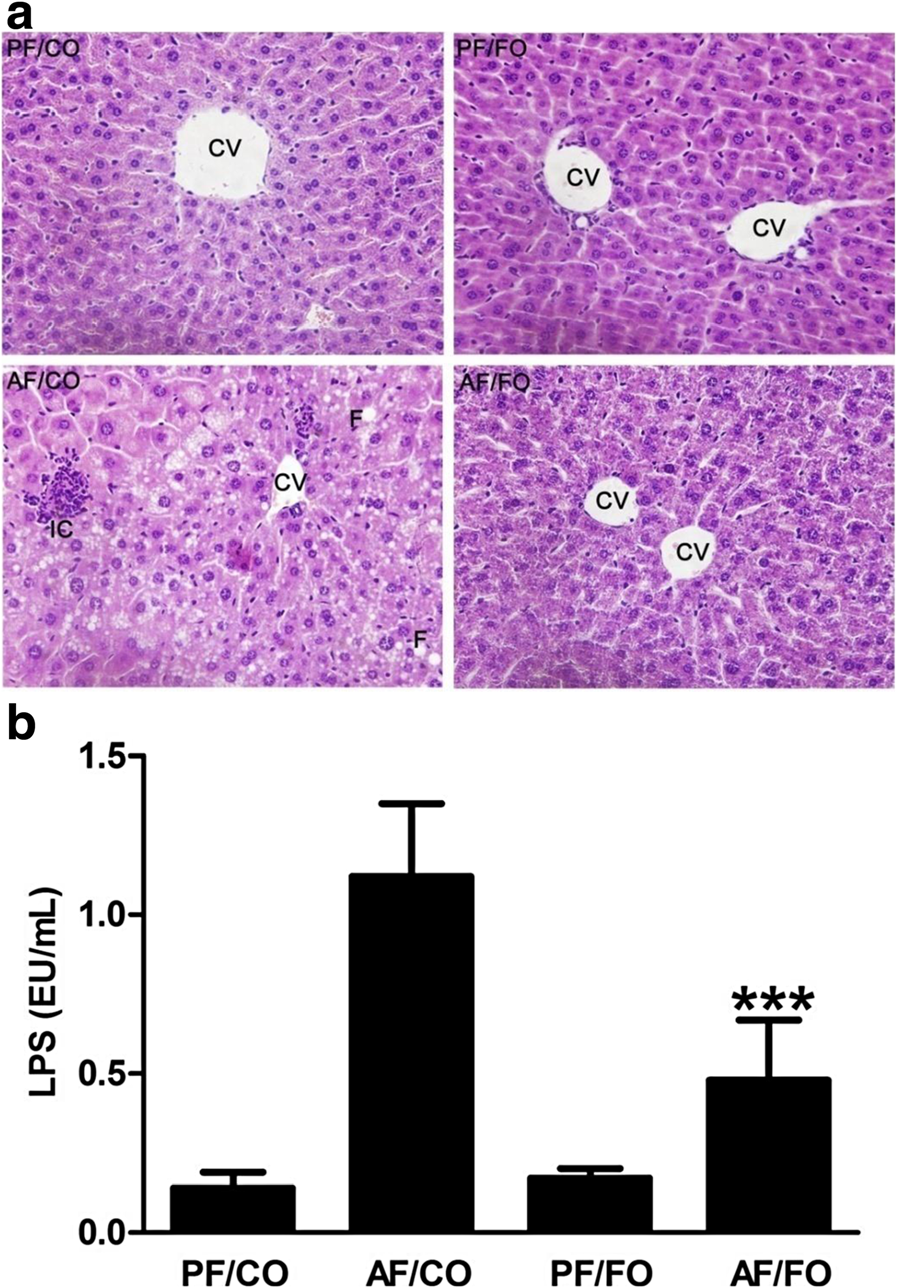
Effects of different dietary oil profile on liver injury and endotoxemia in ALD. **a**: Representative images of hepatic hemaatoxylin and eosin (H&E) staining. **b**: Plasma lipopolysaccharide (LPS) levels. Data are expressed as mean ± SEM. *P < 0.05, **P < 0.001, ***P < 0.0001. Original magnification, ×200 (A). CV, central vein; F, fatty change; IC, inflammatory cells

According to HE staining for liver in diverse groups, hepatic fatty change, necrosis and inflammation were serious in chronic alcohol feeding group (AF/CO), whereas long-term dietary FO distinctly alleviated the alcohol-induced hepatic histopathological injury (Fig. 1a).

Plasma LPS in AF/FO group was significantly decreased compared with AF/CO group (*P* < 0.0001), but still higher than PF/CO or PF/FO group (Fig. 1b), demonstrating that dietary FO possessed ability to attenuated LPS generation from Gram-negative pathogenic bacteria.

### Dietary FO reduced plasma inflammatory cytokine levels in ALD

**Fig. 2:**
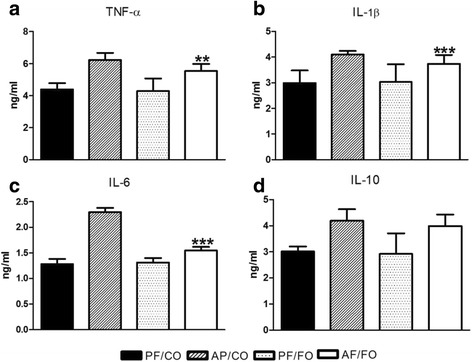
Detection of plasma inflammatory cytokine levels from diverse groups in mice. Plasma of mice from diverse groups were collected respectively for detection of TNF-α (**a**), IL-1β (**b**), IL-6 (**c**) and IL-10 (**d**) concentrations using ELISA kit. Data are expressed as mean ± SEM.*P < 0.05, **P < 0.001, ***P < 0.0001

After chronic ethanol feeding, we found obvious elevated plasma TNF-α, IL-1β, IL-6 and IL-10 in AF/CO and AF/FO groups compared with these cytokines in pair-fed group (Fig. 2). However, dietary FO attenuated ethanol-inducing abnormal elevated TNF-α concentration, compared with that in PF control group (*P* = 0.0095, Fig. 2a). Similarly, plasma IL-1β (*P* = 0.007, Fig. 2b) and IL-6 (*P* < 0.0001, Fig. 2c) levels in AF/FO were also significantly reduced in comparison with those two cytokines in AF/CO group. It showed no significant difference in plasma IL-10 level between AF/CO and AF/FO groups (*P* = 0.3229, Fig. 2d).

### Dietary FO reduced liver inflammatory cytokine levels in ALD

**Fig. 3:**
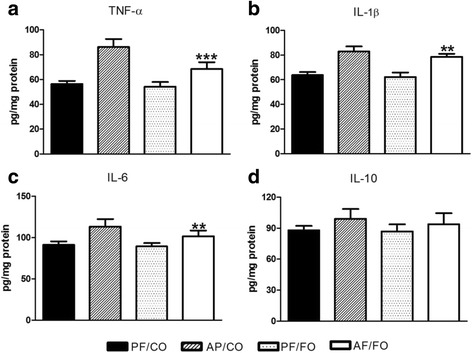
Detection of hepatic inflammatory cytokine levels from diverse groups in mice. Liver tissue of mice from diverse groups were collected respectively for detection of TNF-α (**a**), IL-1β (**b**), IL-6 (**c**) and IL-10 (**d**) concentrations using ELISA kit. Data are expressed as mean ± SEM.*P < 0.05, **P < 0.001, ***P < 0.0001

We detected the cytokine production in liver tissue and also found elevated TNF-α, IL-1β, IL-6 and IL-10 in AF group compared with PF group. Similarly, TNF-α (*p* < 0.001, Fig. 3a), IL-1β (*P* = 0.0021, Fig. 3b) and IL-6 (*P* = 0.0022, Fig. 3c) levels in AF/FO group were significantly decreased compared with those three cytokines in AF/CO group. It showed also no significant difference in IL-10 level in supplementary FO group during chronic ethanol feeding (*P* = 0.1635, Fig. 3d).

### Dietary FO modulated gut microbiota in ALD

Gut microbiota have been increasingly thought to play a critical role in ALD development in mice and humans [3, 14–18]. To investigate whether the observed differences in liver inflammation among AF/CO, AF/FO and those PF groups were associated with the difference in the intestinal microbiota, we performed fecal metagenomic analysis. Rationality of sequencing data was evaluated by rarefaction curve (Additional file 4: Figure S2). It was observed that the rarefaction curve tended to be flat when the sequence number increased to 20,000, indicating that the amount of sequencing data was reasonable.

**Fig. 4:**
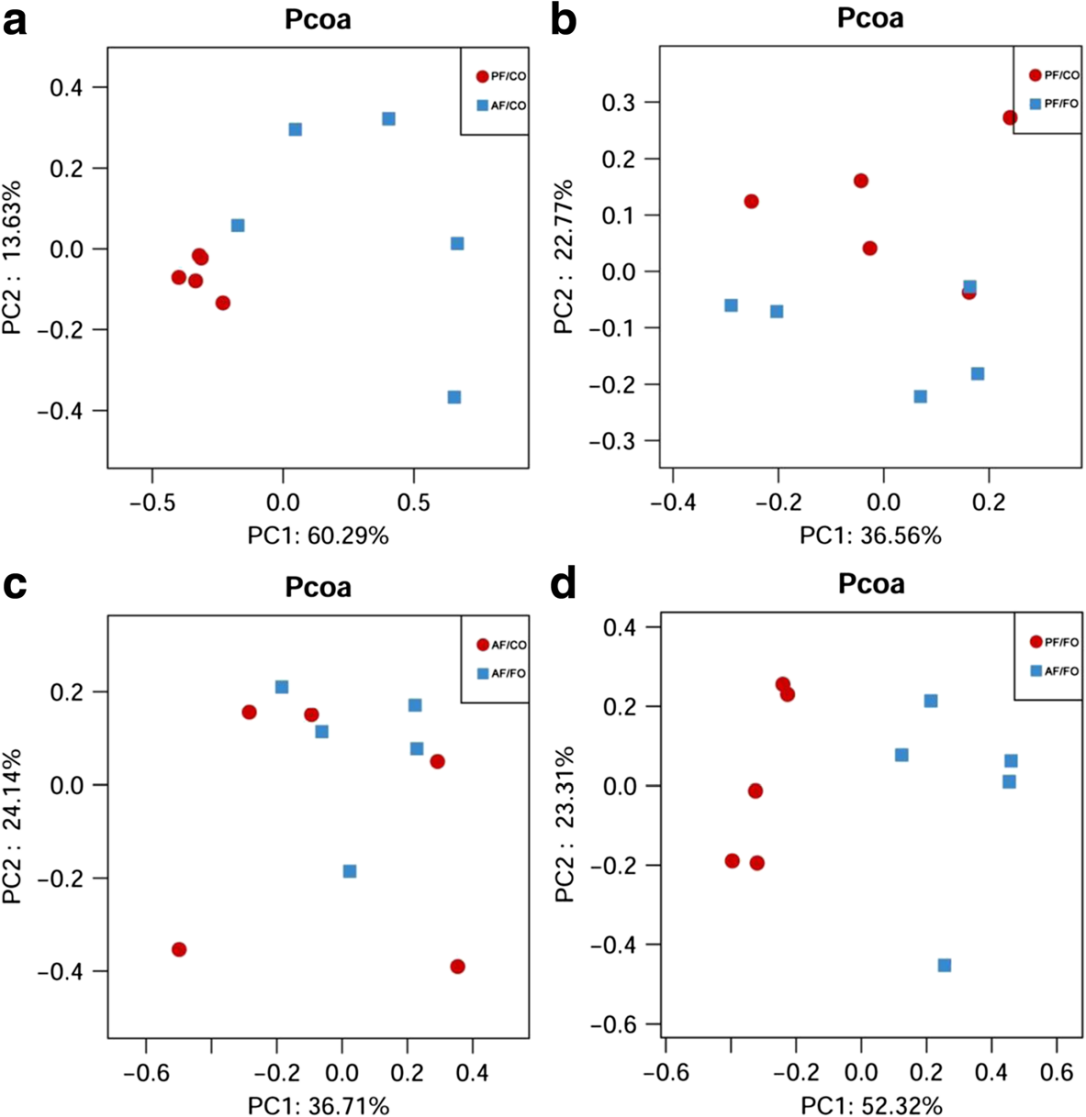
PcoA analysis showing difference in terms of species in fecal samples. Beta diversity was on weighted UniFrac. **a**: PF/CO vs. AF/CO; **b**: PF/CO vs. PF/FO; **c**: AF/CO vs. AF/FO; **d**: PF/FO vs. AF/FO

The overall bacterial community structure was analyzed using unweighted UniFrac (Pcoa) (Fig. 4) and weighted distance matrices (NMDS) (Additional file 5: Figure S3). Pcoa showed that chronic alcohol consumption induced an obvious difference in terms of species in fecal samples compared with pair-fed control feeding (Fig. 4a and b). There’s no obvious change in terms of species between AF/CO group and AF/FO group (Fig. 4c). Interestingly, during normal liquid feeding, supplementary FO seemingly altered the fecal species compared with CO feeding (Fig. 4d). Similar results from NMDS analysis were obtained (Additional file 5: Figure S3).

**Fig. 5:**
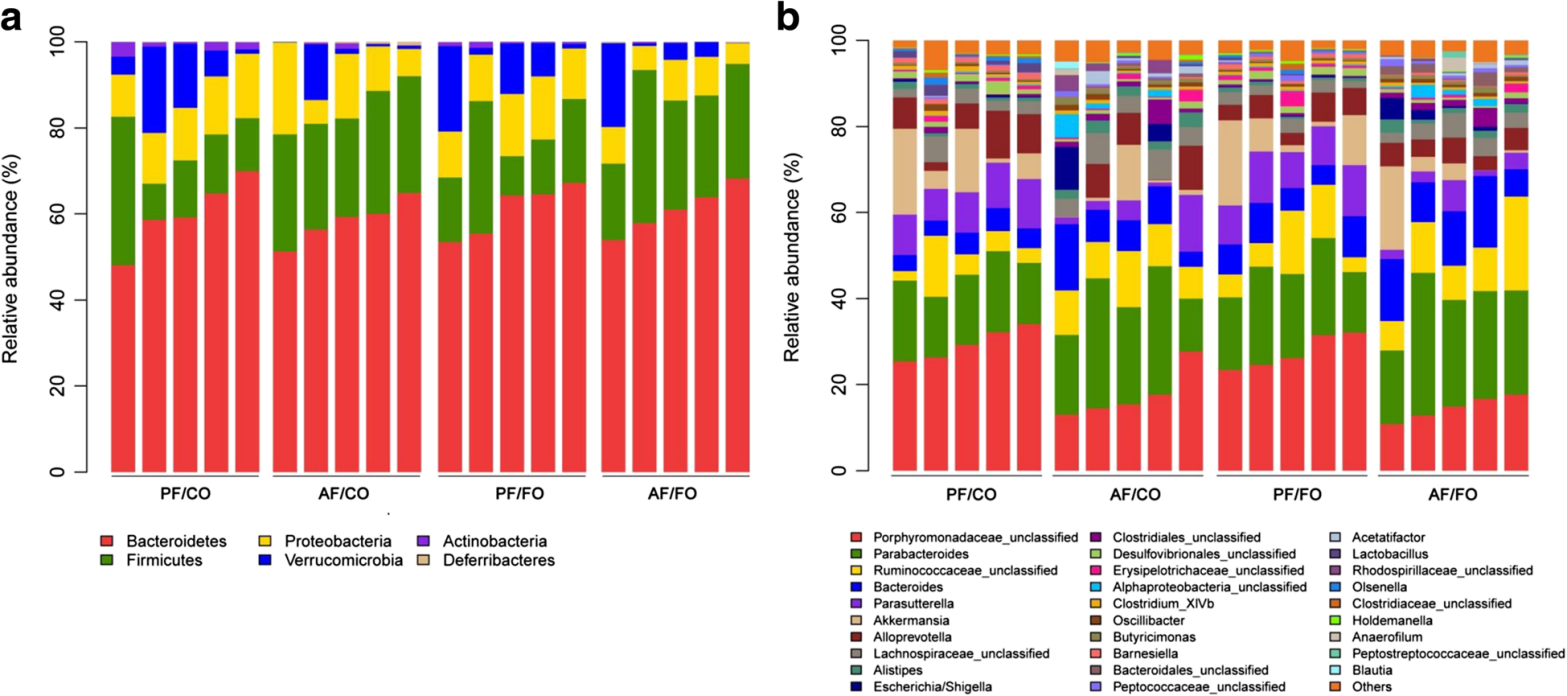
Relative abundance of microbial species at the phylum and genus levels in the feces of mice. **a**: The phylum analysis; **b**: The genus analysis

At phylum level, the proportion of *Firmicutes* was notably increased in alcohol feeding groups compared with those in the PF groups (*P* = 0.0159, Fig. 5a). Meanwhile, there’s no change between AF/FO and AF/CO groups (*P* = 0.8385, Fig. 5a). *Bacteroidetes* accounted for more than half of proportion in diverse administration groups and decreased in AF/CO group in comparison with other three groups but with no significant difference. The proportion of *Proteobacteria* showed no alteration in chronic consumption of alcohol compared with non-ethanol controls. The proportion of *Proteobacteria* in AF/FO group was significantly lower than that in AF/CO group (0.074 ± 0.009 vs. 0.117 ± 0.003, *P* < 0.0001) or PF/FO group (0.074 ± 0.009 vs. 0.124 ± 0.009, *P* < 0.0001). Taken together, our data revealed that under this experimental condition a combination of ethanol and dietary FO (AF/FO) had a major effect on *Proteobacteria* but with limited effects on *Bacteriodetes* and *Firmicutes*.

At genus level, we found *Porphyromonadaceae* was the most prevalent genus in the control groups (PF/CO and PF/FO) and obviously reduced in dietary alcohol administration groups (*P* < 0.0001, Fig. 5b). Moreover, the proportion of *Porphyromonadaceae* in AF/FO group showed lower than that in AF/CO group but without significance (0.176 ± 0.026 vs. 0.146 ± 0.013, *P* = 0.0503). In contrast, *Parabacteroides* was sharply elevated in the AF) groups (AF/CO and AF/FO) compared with the control groups (*P* = 0.0211, Fig. 5b). Additionally, *Parasutterella* was the second prevalent genus in each group. Alcohol administration induced a significant reduction of *Parasutterella* in comparison to that in the control groups (*P* = 0.0005). Collectively, our genus results indicating that chronic alcohol consumption obviously altered the initial proportion of genus components, mainly including *Porphyromonadaceae*, *Parabacteroides* and *Parasutterella*.

**Fig. 6:**
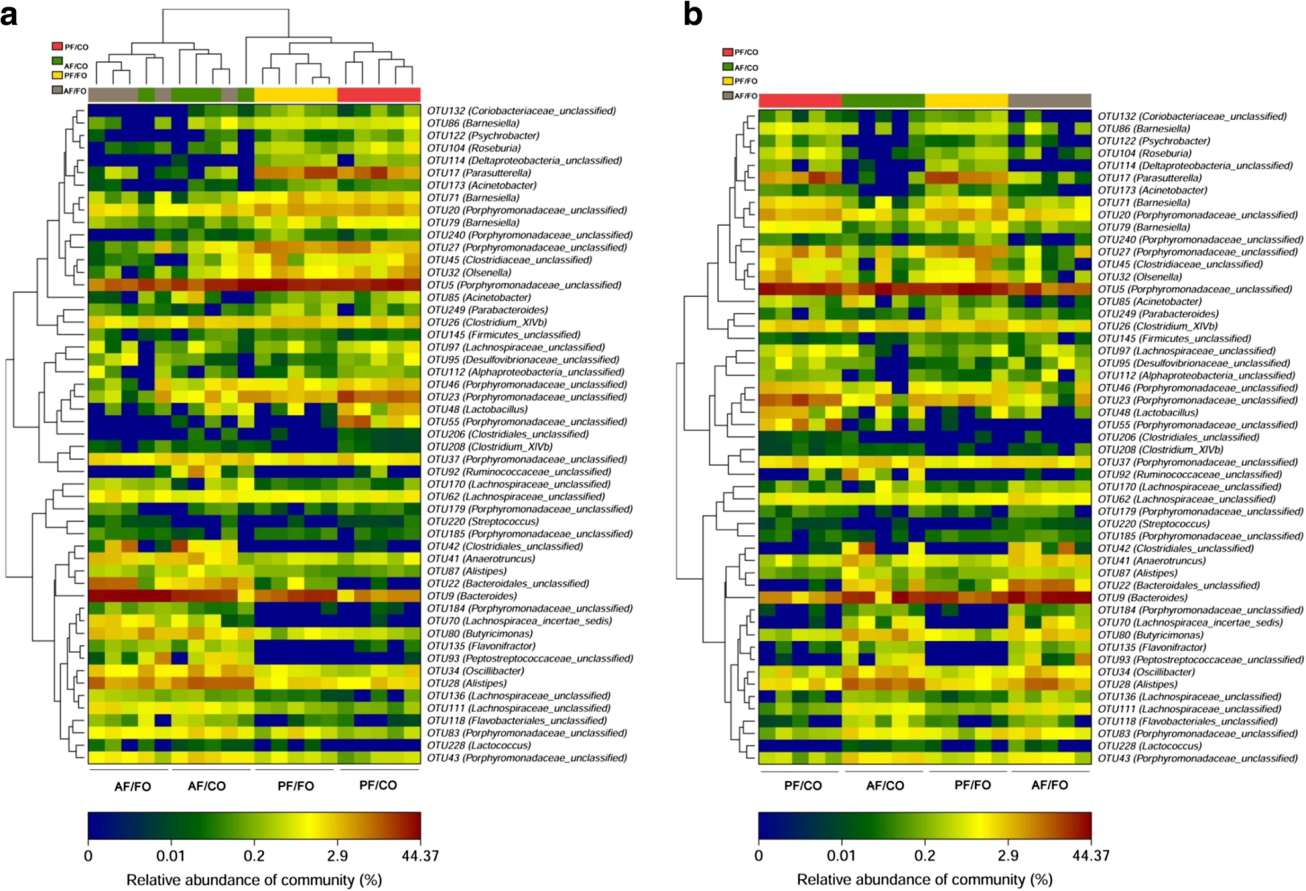
Heatmap analysis of microbial community composition in the feces of mice. **a**: alcohol-fed (AF) vs. pair-fed (PF); **b**: flaxseed oil (FO) vs. corn oil (CO)

Furthermore, heatmap also showed that dietary FO (AF/FO) had a major effect on *Proteobacteria*, with limited effects on *Bacteriodetes* and *Firmicutes*. Moreover, many other tiny bacteria showed obvious difference between AF and PF groups, such as *Barnesiella*, *Psychrobacter*, *Deltaproteobacteria*, *Acinetobacter*, *Flavonifractor*, and *Lactococcus* (Fig. 6a). However, diverse dietary oil had a less effect of on the influence of these seldom bacteria proportion (Fig. 6b).

## Discussion

In the present study, we investigated the efficacy of long-term dietary FO for chronic ALD. By in vivo 6-weeks treatment of ALD in mice, our study demonstrated that supplementary FO showed more effective in reduction of hepatic damage, suggesting that this inexpensive interventions exhibited preventive and therapeutic potential. Our further study revealed that this effective treatment may associated with altered gut microbiota and the decrease of liver inflammation.

Numerous studies indicated that alcohol exposure significantly reduced final BW in chronic ALD [3, 9, 11, 19]. In this study, we also found that BW was lower in AF/CO group, although the caloric intake was identical among all groups. Dietary FO efficiently improved the final BW in ALD compared with AF/CO, indicating that FO may positively affect nutrients absorption and efficiency of calorie utilization in gastrointestinal tract in ALD. Liver weight and relative liver weights in AF group regardless of dietary oil significantly increased, which was consistent with previous reports [9], suggesting that substituting FO for CO in chronic ethanol intake had no effect on liver weight.

In this study, we found abnormal elevated plasma ALT and AST levels in AF/CO group, indicating alcohol induced liver injury [9]. Significant reductions of plasma ALT and AST in AF/FO group revealed that supplementary FO alleviated liver damage caused by chronic ethanol feeding. Similarly, dietary fish oil, rich in long-chain n-3 polyunsaturated fatty acids, mainly eicosapentaenoic acid (EPA) and docosahexaenoic acid (DHA), has showed also the ability to attenuate liver injury by reducing ALT and AST levels in ALD [9, 17]. Inexpensive dietary FO-derived ALA, served as a precursor for the synthesis of EPA and DHA, can converse to EPA and DHA in the blood and tissues [20].

LPS, a trigger for hepatic inflammation in ALD, translocates to liver via portal vein and binds to TLR-4 of antigen presenting cells (APCs) to induce inflammatory immune response and finally cause chronic hepatitis [21, 22]. In this study, plasma LPS in AF/FO group was obviously decreased, demonstrating that dietary FO may decrease gut permeability and reduce LPS translocation from intestines to the liver and systematic circulation in ALD, which contributed to the reduction of inflammatory response in the liver. This attenuation may be associated with intestinal innate immune system and the underlying mechanism needs to be further researched [23].

Activation of Kupffer cells and neutrophils induces oxidative stress and produces inflammatory cytokines, such as TNF-α, IL-1β and IL-6 that cause apoptosis and necrosis of hepatocytes and consequently result in liver injury [9, 24, 25]. Our results showed that TNF-α, IL-1β and IL-6 levels of plasma and liver tissue in AF/FO group were significantly decreased, demonstrating that dietary FO alleviated hepatic inflammation via anti-inflammatory cytokines. IL-10 is an anti-inflammatory cytokine released by Kupffer cells and monocytes [26, 27]. But in this study, we found IL-10 showed no difference among all groups, which was not paralleled with previous study [9]. We speculated that IL-10 maybe play a complicated role in imbalance between regulation of pro- and anti- inflammatory mediators during chronic ethanol exposure. Additionally, regulatory immune cells especially regulatory T lymphocytes (Tregs) [28], which play a critical role in regulation of proinflammation to keep maintain immune balance in ALD [29, 30], need to be investigated in our further study.

Gut micobiota dysbiosis is thought to play a crucial role in the pathogenesis of ALD [6, 31, 32]. In this study, at phylum level, *Bacteriodetes* and *Firmicutes* were the most dominant in all four groups, which were paralleled with previous studies [12, 33]. The proportion of *Firmicutes* was notably increased in alcohol feeding groups compared with the PF groups, which were in agreement with previous studies [3, 32]. Our results showed decreased *Bacteriodetes*and higher *Proteobacteria*in alcohol intake group (AF/CO), which were responsible for gut dysbiosis as recently described in human and animal studies [3, 18]. Importantly, dietary FO notably reduced the proportion of *Proteobacteria* in chronic alcohol consumption, revealing that dietary FO may attenuate gut dysbiosis presumably by modulating gut *Proteobacteria*. Exact mechanism(s) underlying these effects remain to be determined.

At the genus level, decreased gut *Porphyromonadaceae* and inversely elevated *Parabacteroides* were found in chronic alcohol administration. *Porphyromonadaceae* was negatively correlated with TNF-α expression in the liver in ALD [34], which was paralleled with our result and the decrease of gut *Porphyromonadaceae* may benefit for aggravation of the liver inflammation. Elevated *Parabacteroides*in AF/FO group was also involved in the prevention of hepatic inflammation in ALD as previously described [34]. Our results showed that alcohol administration induced a significant reduction of *Parasutterella* in comparison to the control groups. The physiological role of *Parasutterella* is much less understood. Taken together, the exact role of microbiota is complicated and still largely unknown.

## Conclusions

This study highlighted that dietary FO ameliorates alcoholic liver disease via anti-inflammation and modulating gut microbiota in mice, suggesting that it can potentially serve as inexpensive interventions for the prevention and treatment of ALD.

## Additional files

Additional file 1:
**Table S1.** Compositions of the modified Lieber-DeCarli liquid diets. PF/CO, pair-fed with corn oil; AF/CO, alcohol-fed with corn oil; PF/FO, pair-fed with flaxseed oil; AF/FO, alcohol-fed with flaxseed oil (DOCX 12 kb)
Additional file 2:
**Table S2.** Fatty acid composition (%) of dietary fats contained in liquid diets. (DOCX 12 kb)
Additional file 3:
**Figure S1.** Size distribution (predominantly around 20 kb) was estimated by electrophoresis. (DOCX 62 kb)
Additional file 4:
**Figure S2.** Rationality of sequencing data was evaluated by rarefaction curve. It was observed that the rarefaction curve tended to be flat when the sequence number increased to 20,000, indicating that the amount of sequencing data was reasonable. (DOCX 115 kb)
Additional file 5:
**Figure S3.** NMDS analysis showed the difference in terms of species in fecal samples. Beta diversity was analyzed on unweighted Unifrac. A: PF/CO vs. AF/CO; B: PF/CO vs. AF/FO; C: AF/CO vs. AF/FO; D: PF/CO vs. PF/FO. (DOCX 136 kb)
Additional file 6: Datasets for **Figures S1**-**S6**. (ZIP 258 kb)

## Abbreviations

AF: Alcohol-fed
ALA: α-linolenic acid
ALD: Alcoholic liver disease
ALT: Alanine aminotransferase
APCs: Antigen presenting cells
AST: Aspartate aminotransferase
BW: Body weight
CO: Corn oil
DHA: Docosahexaenoic acid
EDTA: Ethylene diamine tetraacetic acid
ELISA: Enzyme linked immunosorbent assay
EPA: Eicosapentaenoic acid
FO: Flaxseed oil
HE: Hematoxylin-eosin
IL: Interleukin
LPS: Lipopolysaccharide
PF: Pair-fed
PUFA: Polyunsaturated fatty acids
TLR-4: Toll-like receptor-4
TNF-α: Tumor necrosis factor
Tregs: Regulatory T lymphocytes
