# Supplementary material for: Flaxseed oil ameliorates alcoholic liver disease via anti-inflammation and modulating gut microbiota in mice
Source: Lipids Health Dis. 2017 Feb 22;16:44. doi: 10.1186/s12944-017-0431-8 (PMC5322643; doi:10.1186/s12944-017-0431-8)
Supplement: Additional file 1: — Table S1. Compositions of the modified Lieber-DeCarli liquid diets. PF/CO, pair-fed with corn oil; AF/CO, alcohol-fed with corn oil; PF/FO, pair-fed with flaxseed oil; AF/FO, alcohol-fed with flaxseed oil (DOCX 12 kb) [file 12944_2017_431_MOESM1_ESM.docx]

**Additional file 1: Table S1.** Compositions of the modified Lieber-DeCarli liquid diets. PF/CO, pair-fed with corn oil; AF/CO, alcohol-fed with corn oil; PF/FO, pair-fed with flaxseed oil; AF/FO, alcohol-fed with flaxseed oil.

| **Ingredients** | **PF/CO** | **AF/CO** | **PF/FO** | **AF/FO** |
| --- | --- | --- | --- | --- |
| Casein, g/L | 41.4 | 41.4 | 41.4 | 41.4 |
| L-cystine, g/L | 0.50 | 0.50 | 0.50 | 0.50 |
| D,L-Methionine, g/L | 0.30 | 0.30 | 0.30 | 0.30 |
| Cellulose, g/L | 10.0 | 10.0 | 10.0 | 10.0 |
| Maltose Dextrin, g/L | 115 | 44.8 | 115 | 44.8 |
| Corn Oil, g/L | 39.6 | 39.6 | - | - |
| Flaxseed Oil, g/L | - | - | 39.6 | 39.6 |
| Mineral Mix (AIN93G-MX), g/L | 8.8 | 8.8 | 8.8 | 8.8 |
| Vitamin Mix (AIN-93-VX), g/L | 2.5 | 2.5 | 2.5 | 2.5 |
| Choline Bitartrate, g/L | 0.53 | 0.53 | 0.53 | 0.53 |
| Vitamine E Acetate, g/L | 0.20 | 0.20 | 0.20 | 0.20 |
| 95% Ethanol (v/v), mL/L | - | 52.6 | - | 52.6 |
