# Supplementary material for: Flaxseed oil ameliorates alcoholic liver disease via anti-inflammation and modulating gut microbiota in mice
Source: Lipids Health Dis. 2017 Feb 22;16:44. doi: 10.1186/s12944-017-0431-8 (PMC5322643; doi:10.1186/s12944-017-0431-8)
Supplement: Additional file 2: — Table S2. Fatty acid composition (%) of dietary fats contained in liquid diets. (DOCX 12 kb) [file 12944_2017_431_MOESM2_ESM.docx]

**Additional file 2: Table S2.** Fatty acid composition (%) of dietary fats contained in liquid diets.

| **Fatty acids** | | **% of total fatty acids** | |
| --- | --- | --- | --- |
| **Common name** | **Symbol** | **Corn oil** | **Flaxseed oil** |
| Lauric acid | 12:0 | 0.2 | 0.2 |
| Myristic acid | 14:0 | 0.9 | 0.7 |
| Palmitic acid | 16:0 | 16.8 | 8.6 |
| Palmitoleic acid | 16:1 n-7 | 0.4 | 0.3 |
| Stearic acid | 18:0 | 4.1 | 5.4 |
| Oleic acid | 18:1 n-9 | 25.7 | 20.2 |
| Linoleic acid (LA) | 18:2 n-6 | 51.0 | 14.3 |
| α-Linolenicacid (ALA) | 18:3 n-3 | 1.0 | 50.3 |
| SFAs |  | 21.8 | 14.9 |
| MUFAs |  | 26.1 | 20.4 |
| Total PUFAs |  | 52.0 | 64.7 |
| n-6 PUFAs/n-3 PUFAs |  | 50.0 | 0.3 |
