# Supplementary material for: Flaxseed oil ameliorates alcoholic liver disease via anti-inflammation and modulating gut microbiota in mice
Source: Lipids Health Dis. 2017 Feb 22;16:44. doi: 10.1186/s12944-017-0431-8 (PMC5322643; doi:10.1186/s12944-017-0431-8)

**Additional file 3: Figure S1.** Size distribution (predominantly around 20 kb) was estimated by electrophoresis.


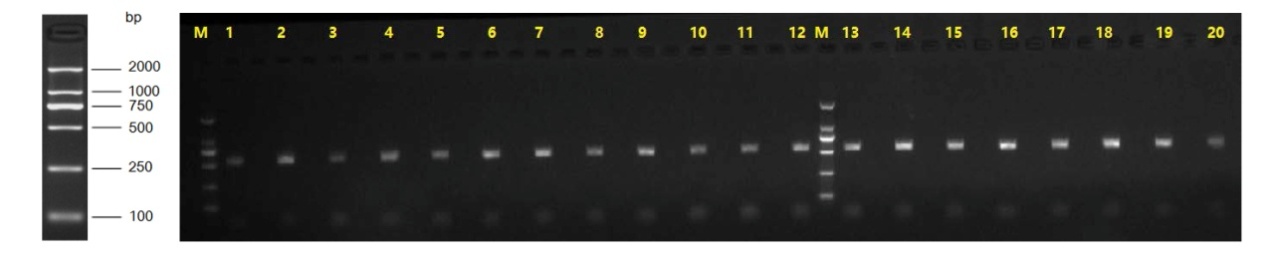

Supplement: Additional file 3: — Figure S1. Size distribution (predominantly around 20 kb) was estimated by electrophoresis. (DOCX 62 kb) [file 12944_2017_431_MOESM3_ESM.docx]
