# Supplementary material for: Flaxseed oil ameliorates alcoholic liver disease via anti-inflammation and modulating gut microbiota in mice
Source: Lipids Health Dis. 2017 Feb 22;16:44. doi: 10.1186/s12944-017-0431-8 (PMC5322643; doi:10.1186/s12944-017-0431-8)

**Additional file 4: Figure S2.** Rationality of sequencing data was evaluated by rarefaction curve. It was observed that the rarefaction curve tended to be flat when the sequence number increased to 20,000, indicating that the amount of sequencing data was reasonable.


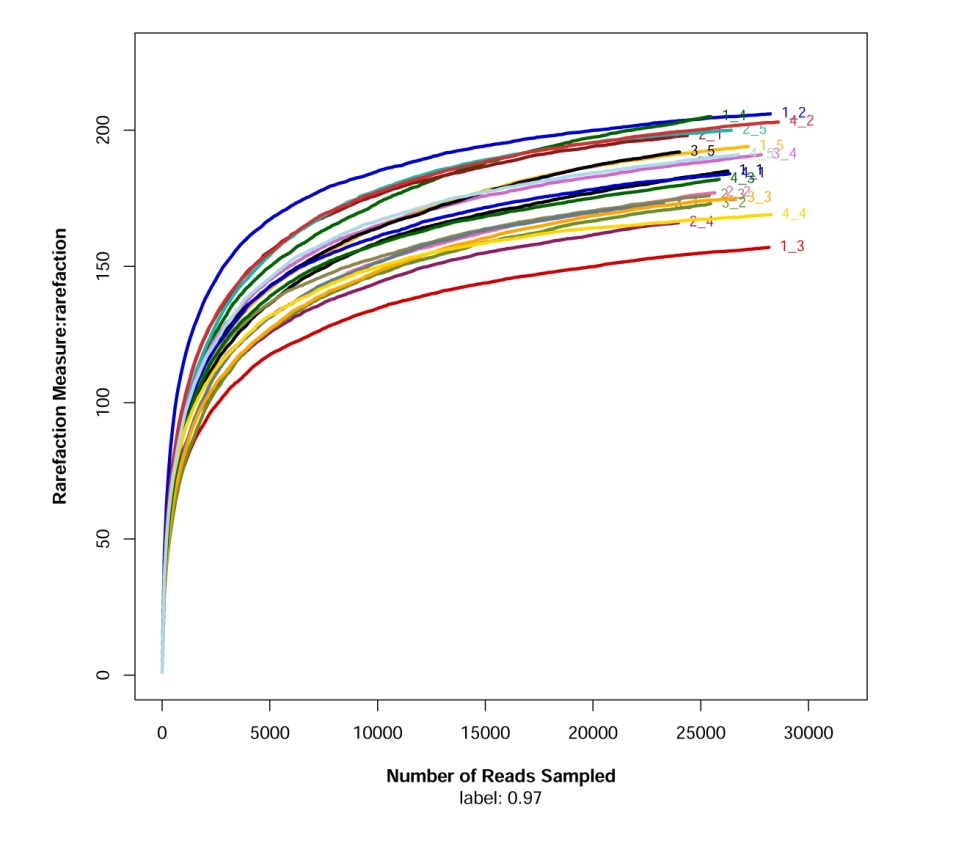

Supplement: Additional file 4: — Figure S2. Rationality of sequencing data was evaluated by rarefaction curve. It was observed that the rarefaction curve tended to be flat when the sequence number increased to 20,000, indicating that the amount of sequencing data was reasonable. (DOCX 115 kb) [file 12944_2017_431_MOESM4_ESM.docx]
