# Supplementary material for: Flaxseed oil ameliorates alcoholic liver disease via anti-inflammation and modulating gut microbiota in mice
Source: Lipids Health Dis. 2017 Feb 22;16:44. doi: 10.1186/s12944-017-0431-8 (PMC5322643; doi:10.1186/s12944-017-0431-8)

**Additional file 5: Figure S3.** NMDS analysis showed the difference in terms of species in fecal samples. Beta diversity was analyzed on unweighted Unifrac. A: PF/CO vs. AF/CO; B: PF/CO vs. AF/FO; C: AF/CO vs. AF/FO; D: PF/CO vs. PF/FO.


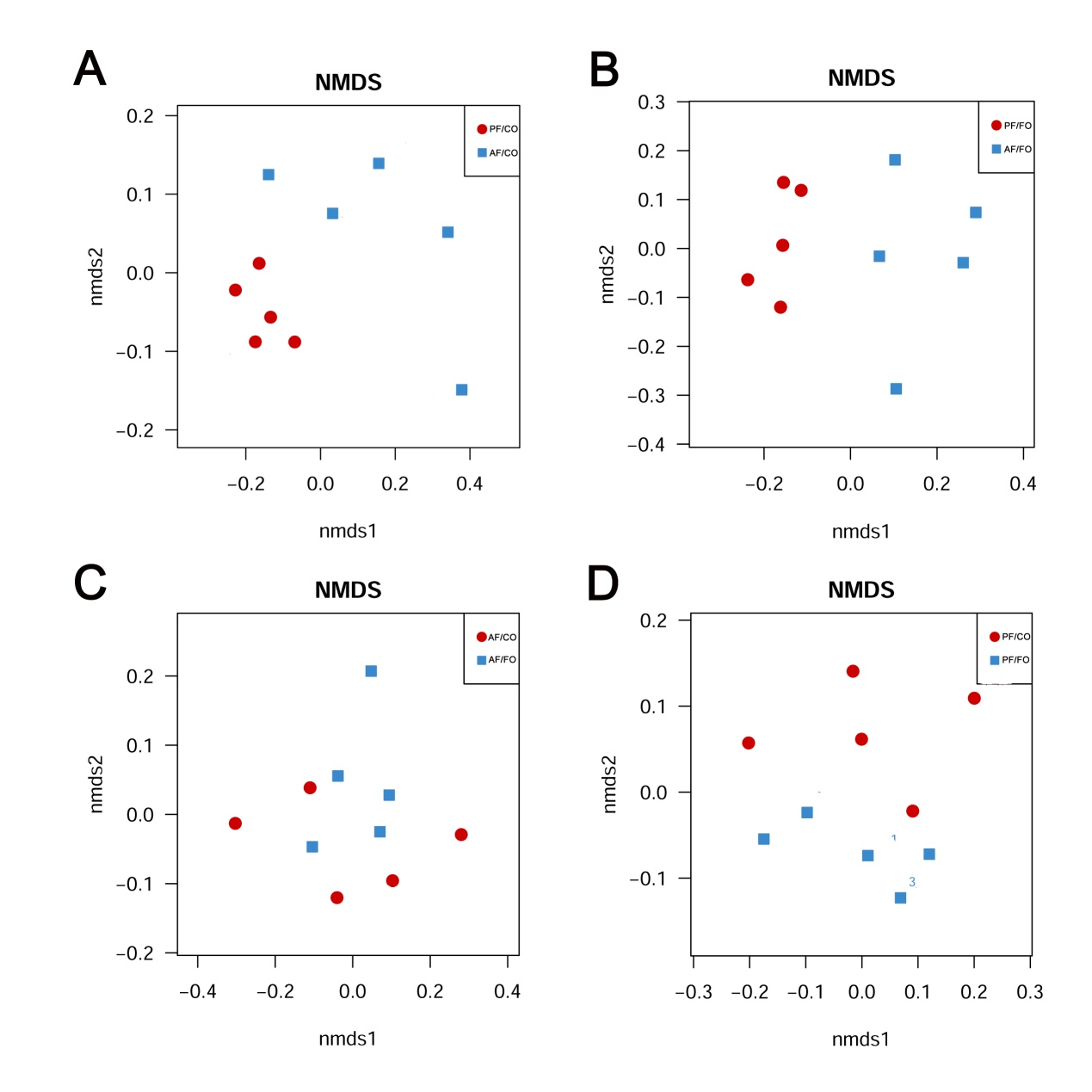

Supplement: Additional file 5: — Figure S3. NMDS analysis showed the difference in terms of species in fecal samples. Beta diversity was analyzed on unweighted Unifrac. A: PF/CO vs. AF/CO; B: PF/CO vs. AF/FO; C: AF/CO vs. AF/FO; D: PF/CO vs. PF/FO. (DOCX 136 kb) [file 12944_2017_431_MOESM5_ESM.docx]
