# Supplementary material for: Flaxseed oil ameliorates alcoholic liver disease via anti-inflammation and modulating gut microbiota in mice
Source: Lipids Health Dis. 2017 Feb 22;16:44. doi: 10.1186/s12944-017-0431-8 (PMC5322643; doi:10.1186/s12944-017-0431-8)
Supplement: Additional file 6: — Datasets for Figures S1-S6. (ZIP 258 kb) [file 12944_2017_431_MOESM6_ESM.zip › Datasets for Fig. 1-3.docx]

1. Data for Fig. 1B.

| Groups | LPS |  |
| --- | --- | --- |
| PF/CO | 0.14 ± 0.05 (n=15) |  |
| AF/CO | 1.12 ± 0.23 (n=15) |  |
| PF/FO | 0.17 ± 0.03 (n=15) |  |
| AF/FO | 0.48 ± 0.19 (n=15) |  |

2. Data for Fig. 2A-D

| Groups | TNF-α | IL-1β | IL-6 | IL-10 |
| --- | --- | --- | --- | --- |
| PF/CO | 4.39 ± 0.39 | 2.99 ± 0.49 | 1.28 ± 0.10 | 3.01 ± 0.19 |
| AF/CO | 6.23 ± 0.44 | 4.10 ± 0.14 | 2.30 ± 0.08 | 4.19 ± 0.44 |
| PF/FO | 4.29 ± 0.79 | 3.03 ± 0.69 | 1.31 ± 0.09 | 2.92 ± 0.79 |
| AF/FO | 5.54 ± 0.61 | 3.74 ± 0.34 | 1.55 ± 0.07 | 3.99 ± 0.44 |

3. Data for Fig. 3A-D

| Groups | TNF-α | IL-1β | IL-6 | IL-10 |
| --- | --- | --- | --- | --- |
| PF/CO | 56.29 ± 2.37 | 63.70 ± 2.32 | 90.88 ± 4.11 | 88.14 ± 4.22 |
| AF/CO | 87.13 ± 6.24 | 82.90 ± 4.71 | 113.25 ± 9.05 | 99.26 ± 9.51 |
| PF/FO | 53.17 ± 3.65 | 62.20 ± 3.63 | 89.27 ± 4.02 | 86.87 ± 6.13 |
| AF/FO | 68.40 ± 5.44 | 78.71 ± 2.33 | 101.51 ± 7.12 | 94.03 ± 10.29 |
